# Differential depletion of total T cells and regulatory T cells and prolonged allotransplant survival in CD3Ɛ humanized mice treated with polyclonal anti human thymocyte globulin

**DOI:** 10.1371/journal.pone.0173088

**Published:** 2017-03-03

**Authors:** Maja Buszko, Benno Cardini, Rupert Oberhuber, Lukas Oberhuber, Bojana Jakic, Anja Beierfuss, Georg Wick, Giuseppe Cappellano

**Affiliations:** 1 Laboratory of Autoimmunity, Division of Experimental Pathophysiology and Immunology, Biocenter, Medical University of Innsbruck, Innsbruck, Austria; 2 Department of Visceral, Transplant and Thoracic Surgery, Center of Operative M edicine, Medical University of Innsbruck, Innsbruck, Austria; 3 Central Laboratory Animal Facility, Medical University of Innsbruck, Innsbruck, Austria; Mie Daigaku, JAPAN

## Abstract

Thymoglobulin (ATG) is a polyclonal rabbit antibody against human thymocytes used as a T cell-depleting agent to prevent or treat allotransplant rejection. The aim of the present study was to investigate the effect of low dose ATG treatment exclusively on T cells using a humanized BALB/c human CD3Ɛ transgenic mouse model expressing both human and murine T cell receptors (TCR). Mice received a single intravenous (i.v.) injection of ATG. Blood and peripheral lymphoid organs were obtained after different time points. We found a significant T cell depletion in this mouse model. In addition, regulatory T cells (Tregs) proved to be less sensitive to depletion than the rest of T cells and the Treg:non-Treg ratio was therefore increased. Finally, we also investigated the effect of ATG in a heterotopic allogenic murine model of heart transplantation. Survival and transplant function were significantly prolonged in ATG-treated mice. In conclusion, we showed (a) an immunosuppressive effect of ATG in this humanized mouse model which is exclusively mediated by reactivity against human CD3Ɛ; (b) provided evidence for a relative resistance of Tregs against this regimen; and (c) demonstrated the immunomodulatory effect of ATG under these experimental circumstances by prolongation of heart allograft survival.

## Introduction

Thymoglobulin (ATG) is a solution of rabbit anti-human thymocytes immunoglobulin. It has been used as an immunosuppressive agent in the prevention and treatment of transplant rejection such as kidney, liver and heart for decades [1,2]. The immunosuppressive effects of ATG are based on complement-mediated cell lysis and apoptosis [3].

In the field of allotransplantation, another antibody-based immunosuppressive therapy specifically targets CD3 molecules [4]. The main effect of anti-CD3 monoclonal antibodies (mAbs) (OKT3) is T cell depletion [5]. In contrast to OKT3 antibodies (Abs), the mode of action of ATG is more complex since Ab specificities comprise molecules expressed not only by T cells but also by other cells of other hematopoietic origin such as B cells, monocytes, NKT cells as well as cells of non-hematopoietic lineage, like endothelial cells [2]. This polyreactivity implies a very complicated mode of action of ATG. Indeed, in humans, the difficulty in assessing the effect of ATG on different cell populations lies in the limited access to lymphoid organs. Only one study performed on nonhuman primates showed that the dose as well as the timing of ATG application determines the outcome on T cell depletion in peripheral lymphoid organs, with the highest dose applied before the transplant procedure being the most potent (clearing up to 85% of T cells in peripheral lymphoid organs without affecting the thymus) [3].

Moreover, the mechanism of OKT3’s action relies exclusively on the specific interaction with the epsilon (Ɛ) chain of the CD3 protein in association with the T cell receptor (TCR) complex. In contrast, as quantified by Popow et al., the ATG used in this study contains only 0.283 μg/ml of anti-CD3 Abs, which suggests a combination of different mechanisms involved in its immunosuppressive effect [6].

In murine research, a monoclonal anti-mouse CD3Ɛ mAb (clone 145 2C11) is used as a surrogate for OKT3 [7,8] and it has been shown that *in vivo* administration of high doses of this mAb prolongs transplant survival [7]. In contrast, low doses of 145-2C11 reversed spontaneous diabetes in NOD mice [8] and decreased lipid accumulation in LDLr^-/-^ mice, thus inhibiting atherosclerosis progression [9]. Furthermore, it has been suggested that in spite of overall depletion of pathogenic effector T cells [9], OKT3 may spare regulatory T cells (Tregs) [10].

Helios is a transcription factor that controls differentiation, suppressive activity and survival of Tregs [11]. The Fc non-binding anti-mouse CD3Ɛ mAb was shown to influence the expression of the transcription factor Helios in Tregs and thus positively affecting the Treg:non-Treg balance [10]. In addition, it was suggested that tolerance induction, after treatment with anti-mouse CD3Ɛ mAb, is based on mechanisms that are TGF-β dependent [12].

To investigate the action of human ATG, polyclonal rabbit anti-mouse thymocyte globulin (mATG) has been used as surrogate [13–15]. The experimental results showed that mATG depletes T cells in blood, spleen, lymph nodes, and bone marrow but not in the thymus. Finally, as shown for OKT3, a lesser depletion was observed for both Tregs and memory T cells after mATG treatment [15,16].

Currently, humanized mice represent a very useful tool in animal model research and enable translating basic knowledge to be used in human patients. Human CD3Ɛ expressing mice were employed for studying TCR composition and function [17] as well as the therapeutic potential using OKT3 Abs for diabetes [18].

In this mouse model, huCD3Ɛ expression is driven by the CD2 promoter and the resulting TCRs contain both mouse and huCD3 receptors [17]. We used this model to investigate the main effect of ATG on the human CD3Ɛ receptor in order to scrutinize the immunomodulatory actions mediated via this pathway.

In this study, we investigated the effect of ATG while limiting its multiple targets to T cells exclusively. We found that a single intravenous (i.v.) injection of ATG into BALB/c huCD3Ɛ transgenic mice resulted in T cell depletion in the circulation and in the secondary lymphoid organs, with concomitant preservation of Tregs. The outcome of the process altered Treg:non-Treg ratio that may play an additional role in the immunosuppressive effect of ATG. Moreover, we demonstrated a prolongation of heart allograft survival.

## Materials and methods

### Ethics statement

Mice were maintained under Specific Pathogen Free (SPF) conditions and professional routine health monitoring was performed quarterly. All experimental procedures were conducted according to the Austrian Animal Welfare Law and were approved by the Federal Austrian Ethics Committee for Animal Experimentation (approval number: BMWFW-66.011/0066-WF/II/3b/2014). Animal welfare was monitored at least daily. Mice were euthanized by CO_2_ inhalation followed by cervical dislocation.

In heart allotransplant experiments, mice were anesthetized by intraperitoneal injection of 5mg/kg body weight of Xylazine and 100mg/kg body weight of Ketamine. In order to minimize animals' stress and suffering, Buprenorphin (0.1 mg/kg body weight) was administered subcutaneously (s.c.) right after the operation and every 12 hr for 3 days. In addition, carprofen (4mg/kg) was administered s.c. every 12 hr for 7 days. Animal health status, welfare and its water uptake were monitored at least twice a day. If there was evidence of more than 15% weight loss compared to weight at surgery-date, no food intake, breathing difficulties, apathy, crippling or a hunched position as well as when the heart was rejected, animals were euthanized by terminal isofluran inhalation followed by cervical dislocation. Two unexpected deaths were caused by thromboembolic events early after surgery.

### Mice

BALB/c-huCD3Ɛ (*H-2*^*b*^) transgenic mice were kindly provided by Professor Lucienne Chatenoud (INSERM U1151, Paris, France). BALB/c huCD3Ɛ, BALB/c (*H-2*^*b*^) and C57BL/6 (*H-2*^*d*^) wt mice were bred and maintained in Central Laboratory Animal Facilities of Medical University of Innsbruck. Female mice were used unless otherwise indicated. All mice were used at 8–14 weeks of age.

### ATG or control rabbit immunoglobulin (Ig) administration

Human ATG was provided by Sanofi Aventis (Genzyme Paris) and normal control rabbit Ig was purchased from Dianova (Hamburg, Germany). Both infusions were prepared accordingly to the manufacturer's instructions. A single dose of 100 μg of ATG or rabbit Ig diluted in 100 μl PBS was i.v. injected into the tail vein and the proportion of T cells was assessed at the indicated time points.

### Reagents and antibodies

RPMI-1640 medium was purchased from Lonza (Walkersville, MD) and complete culture media were prepared using RPMI-1640 containing 10% fetal bovine serum (FBS) (GE healthcare, Piscataway, NJ), 50 μM β-mercaptoethanol, 2 mM glutamine and 100 U/ml of penicillin and streptomycin (Lonza). Anti-mouse CD3 mAb (clone: 145 -2C11) was produced in-house or purchased from R&D (Minneapolis, MN). Anti-human CD3 mAb (OKT3), anti-mouse CD4 (RM4-5), anti-mouse CD25 (PC61.5), anti-mouse CD8α (53–6.7), anti-mouse Foxp3 (FJK-16s), anti-mouse/human Helios (22F6), anti-Armenian hamster IgG and anti-rabbit IgG were purchased from eBioscience (San Diego, CA). The carboxyfluorescein succinimidyl ester (CFSE) was purchased from Molecular Probes (Eugene, Oregon). Mouse IFN-ɣ ELISA kit was purchased from eBioscience.

### Cells preparation from different tissues

At indicated times, mice were sacrified and blood, spleen, lymph nodes (inguinal, brachial and axillary) and thymus were harvested. Single cell suspensions were generated from spleen, lymph nodes and thymus by homogenization using a 100 μm cell strainer on a petri dish. Erythrocytes were lysed with lysis buffer for 5 min and then the cells were washed with PBS containing 3% FBS. Cells were washed again with PBS containing 3% FBS or with complete medium before use in the assays described below. Viable cells were counted by trypan blue exclusion (Sigma-Aldrich, St.Louis, MO).

### Flow cytometry

For flow cytometric analysis cells were stained 20–30 min at 4°C in the dark. For intracellular staining of Foxp3 and Helios, cells were permeabilized for 45 min with Fix/Perm buffer (eBioscience) and then washed with permeabilization buffer. Anti-Foxp3 and anti-Helios Abs were added and the cells incubated for another 30 min according to the instructions of the manufacturer (eBioscience). Flow cytometric analysis was performed on a FACSCalibur (BD, San Jose, CA) and data were analyzed using FlowJo software (Tree Star Inc., Ashland,OR).

Absolute cell numbers were calculated by multiplying the percentage of each cell population within the specific gates by the total cell number counted by trypan blue exclusion.

### In vitro stimulation assay

2 x 10^5^ splenocytes labeled with 2 μM CFSE were stimulated with plate-bound anti-mouse or human CD3, or ATG in complete media. Cells were incubated at 37°C, 95% humidity and 5% CO_2_ for 3 days. Cell division was assessed by analysis of the CFSE dilution.

### Murine cervical heart transplantation

Male mice were first anesthetized by intramuscular (i.m.) injection of xylazine (5 mg/kg body weight) and ketamine (100 mg/kg body weight). Then, a single dose (100 μg) of ATG or control rabbit Ig was administered by i.v. injection prior to surgery. C57BL/6 (H-2^b^) mice were used as heart donors.

Donor grafts were transplanted heterotopically into the recipients’ neck using a modified cuff technique [19]. Graft function was evaluated daily accordingly to the heart beat score evaluation as reported in [20], scoring system: 0: no organ function, 1: fibrillation, only visible through magnification, 2: poor or partial organ function, 3: impairment in frequency or intensity of heart beating, 4: physiological organ function.

### Data analysis

Data are represented as mean ± standard error of the mean. Differences observed between the two groups were determined using the Mann-Whitney test (GraphPad Software, San Diego, CA). Differences with *p<0*.*05* were considered statistically significant.

## Results

### Binding of ATG to CD3Ɛ and in vitro stimulation

**Fig 1:**
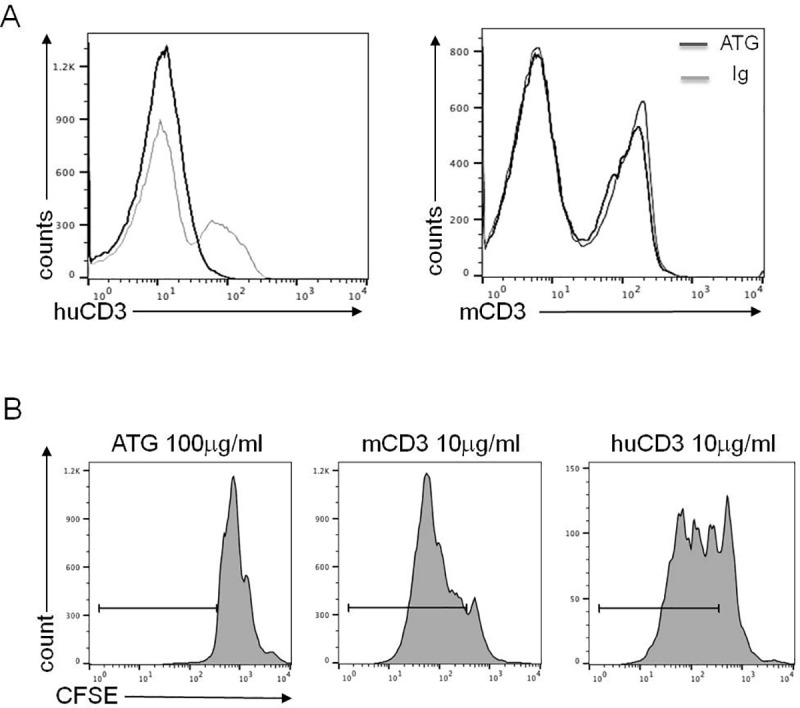
*In vitro* effect of ATG on BALB/c huCD3Ɛ splenocytes. (A) Binding of ATG to CD3Ɛ. BALB/c huCD3Ɛ splenocytes were cultured for 24 h with ATG or control Ig and then stained with anti-huCD3 or anti-mouse CD3 mAbs. Staining was assessed by FACS. (B) Proliferation of CFSE-labeled BALB/c huCD3Ɛ splenocytes after 3 days stimulation with plate-bound ATG, anti-mouse CD3 or anti-huCD3 mAbs, marked by solid line. Representative data of at least 2 independent experiments are shown.

To assess the binding of ATG to CD3Ɛ *in vitro* on splenocytes of BALB/c huCD3Ɛ mice, we performed FACS staining of cells incubated previously with ATG or control rabbit immunoglobulin (Ig). By staining with anti-mouse and anti-huCD3Ɛ mAbs we showed that there was a complete loss of huCD3Ɛ receptor staining after incubation with ATG as compared to control Ig. In contrast, no decrease in the staining of mCD3 was observed (Fig 1A). We then examined the potential of ATG to induce proliferation *in vitro*. Splenocytes were isolated and incubated with plate-bound ATG, anti-huCD3Ɛ or anti-mCD3Ɛ mAb for 3 days. As shown by CFSE dilution, we detected proliferation using anti-mouse CD3Ɛ mAbs or anti-human-CD3Ɛ mAbs. However, the murine splenocytes did not proliferate after stimulation with ATG in doses ranging from 1 to 100 μg/ml (Fig 1B and data not shown). These data show the limited potential of *in vitro* stimulation by ATG.

### Intravenous injection of ATG in BALB/c huCD3Ɛ mice depletes T cells

**Fig 2:**
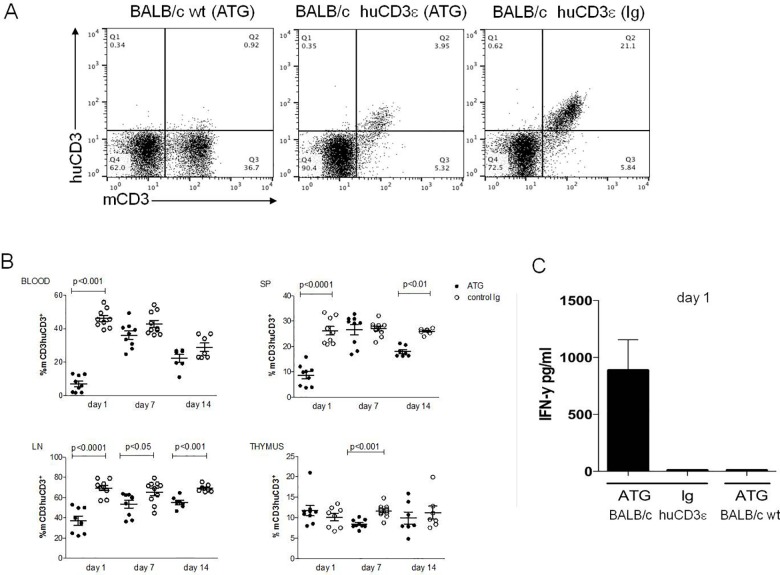
Depletion of T cells after ATG treatment. BALB/c wt or BALB/c huCD3Ɛ were injected i.v with ATG or control rabbit Ig. Cells were stained with anti-human and anti-mouse CD3 mAbs and depletion of T cells was assessed by FACS in blood, spleen (SP), lymph nodes (LN) and thymus at the given time points. (A) Representative FACS plots of stained splenocytes. (B) Dot plots showing mCD3 and huCD3 staining as % lymphocyte. Six to nine mice per group (each data point represents an individual mouse), 2–3 independent experiments were performed. Mann-Whitney statistical test was used. Data are shown as means ± SEM. C. Serum IFN-ɣ levels after injection of ATG were measured by ELISA, 3–6 mice are shown.

To evaluate the depleting capacity of ATG *in vivo*, we assessed the proportion of CD3^+^ T cells 24 h, 7 and 14 days after single i.v. injection of ATG (100μg) in BALB/c huCD3Ɛ and wild type (wt) BALB/c mice (Fig 2A). As shown in Fig 2B, we observed that after 24 h ATG significantly depleted CD3^+^ T cells in blood, as compared to mice injected with control rabbit Ig and with PBS (data not shown). Moreover, we found a significant depletion in spleen and in lymph nodes. Although after 7 and 14 days, the number of T cells recovered in the circulation, a significant decrease was still observed in spleen (day 14) and lymph nodes (day 7 and 14). Additionally, we found that ATG depleted T cells in the thymus as observed on day 7 (Fig 2B). The depletion was reflected in the absolute number (S1A Fig). T cell depletion persisted in blood, lymph nodes and spleen 35 days after injection (S2A Fig). Moreover, ATG did not deplete B cells (data not shown). ATG-mediated depletion did not occur in wt BALB/c mice (Fig 2A).

Following ATG treatment, a “cytokine storm” has occasionally been observed in patients. IFN-ɣ, a hallmark of T cell activation is one of the most abundant cytokines induced by ATG treatment [21] as well as in OKT3-treated patients [22]. In addition, in NOD huCD3Ɛ mice it has been shown that a single i.v. dose of anti-mouse CD3 mAbs induced the release of IFN-ɣ [18]. In line with these clinical and experimental observations, we found a significant amount of IFN-ɣ levels in the serum of BALB/c huCD3Ɛ mice, detectable only 24h after injection (Fig 2C).

Since assessment of lymphocyte levels outside the circulation has not been possible in humans, our results obtained in BALB/c huCD3Ɛ mice suggest that long-lasting T cell lymphopenia may also occur in peripheral lymphoid organs after ATG treatment.

### ATG affects CD4:CD8 ratio and preferentially depletes naïve T cells

**Fig 3:**
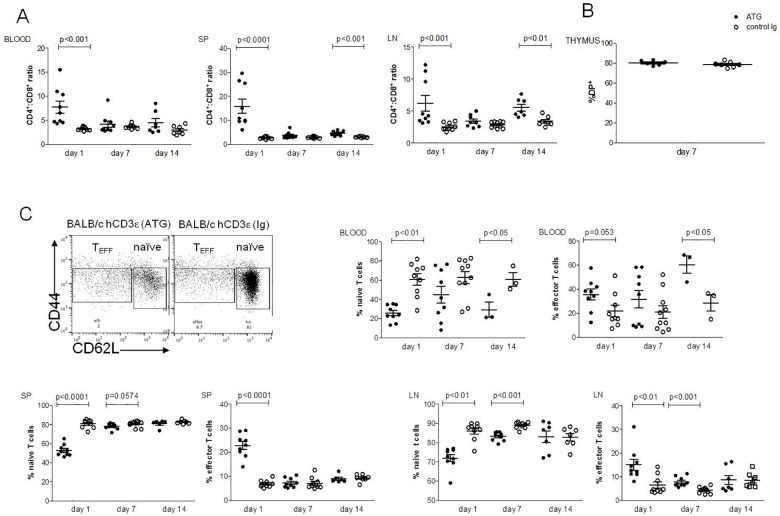
Depletion of T cell subsets after ATG treatment. BALB/c huCD3Ɛ were injected i.v with ATG or control rabbit Ig. Cells were stained and depletion of T cells was assessed by FACS in blood, spleen (SP), lymph nodes (LN) and thymus, at the given time points. (A) Cells were gated on huCD3^+^ cells, and CD4:CD8 ratios are shown in different organs. (B) CD4^+^CD8^+^ double positive (DP), T cells in the thymus, shown as % lymphocytes. (C) Cells were gated on CD4^+^ cells, and naïve and effector T cells were determined and shown as % CD4+. Six to nine mice per group (each data point represents an individual mouse), 2–3 independent experiments were performed. Data are shown as means ± SEM. Mann-Whitney statistical test was used.

To investigate which T cell subset was most affected by ATG, we first determined its effects on CD4^+^ and in CD8^+^ T cells in all lymphoid organs mentioned above. As shown in S1B Fig, we found a significant decrease of both CD4^+^ and CD8^+^ T cells in spleen and in lymph nodes compared to mice injected with control rabbit Ig. Interestingly, in ATG-treated mice, the CD4:CD8 ratio was significantly higher in the blood, spleen and lymph nodes, compared to the mice injected with control rabbit Ig (Fig 3A and S2B Fig). These results suggest that CD8^+^ T cells are more sensitive to ATG-mediated depletion than CD4^+^ T cells.

Although a significant T cell depletion after 7 days in the thymus was observed (Fig 2B), there was no depletion of CD4^+^CD8^+^ double-positive (DP) T cells (Fig 3B) as seen after anti-mouse CD3 mAb treatment [23]. Moreover, we found that percentage and absolute number of naïve CD4^+^ cells were significantly lowered in spleen and lymph nodes 24 h after i.v. injection of ATG as depicted in Fig 3C and in S1B Fig. T cell depletion still persists in spleen and lymph nodes after 7 days. By contrast, effector T cells were less sensitive to ATG-mediated depletion (Fig 3C).

### Tregs are relatively resistant to ATG mediated T cell depletion

**Fig 4:**
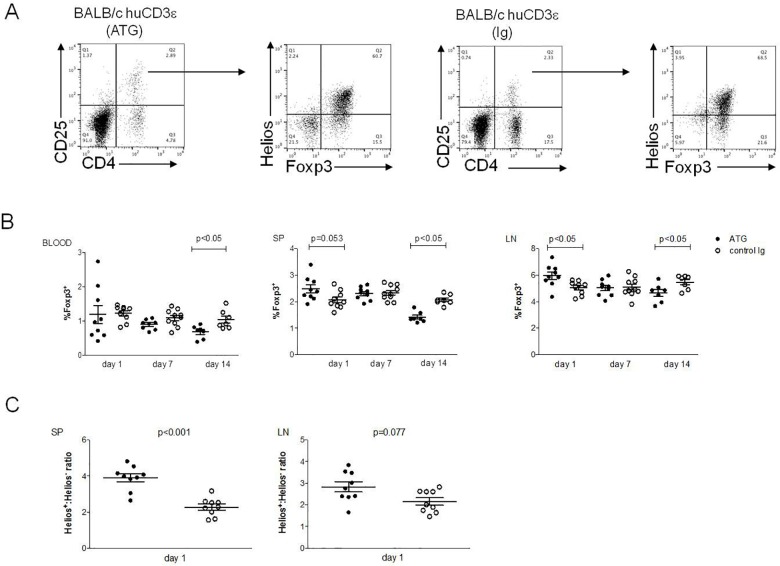
Relative resistance of Tregs after ATG treatment. BALB/c huCD3Ɛ were injected i.v with ATG or control rabbit Ig. Tregs were stained and their frequencies were assessed by FACS in different organs at the given time points. (A) Representative FACS plots showing gating strategy in analysis of Foxp3 and Helios expression in spleen. (B) Frequencies of Foxp3^+^ Tregs shown as % lymphocytes. (C) Helios^+^: Helios^-^ Foxp3^+^ ratios in the spleen (SP) and lymph nodes (LN) 1 day post injection. Six to nine mice per group (each data point represents an individual mouse), 2–3 independent experiments were performed. Data are shown as means ± SEM. Mann-Whitney statistical test was used.

It has been shown that anti-CD3Ɛ mAbs administration selectively depletes non-Tregs, potentially by increasing the stability of Tregs [10]. Although we found that at 24 h post injection, most CD3^+^ T cells were depleted (Fig 2A and 2B), Tregs were, interestingly, shown to be relatively resistant to ATG-mediated depletion (Fig 4A and 4B). We observed this phenomenon in peripheral blood, spleen and lymph nodes as represented by relative frequencies as well as in absolute numbers (S1C Fig). In addition, in lymph nodes and, marginally, in the spleen, the proportion of Tregs within the total lymphocyte population was significantly increased. The resistance of Tregs to the ATG-mediated T cell depletion resulted in a clear-cut increase of the Treg:non-Treg ratio in secondary lymphoid organs (Fig 4C). Moreover, the proportion of CD4^+^CD25^+^ cells also increased 24 h after ATG injection in spleen and lymph nodes, but this increase was most probably due to an upregulation of CD25 by non-Tregs (Fig 4A). However, 14 and 35 days after ATG treatment, Tregs also seemed to be slightly decreased in the lymph nodes (Fig 4B and S2C Fig). These decreased frequencies were not reflected by absolute numbers (S1C Fig).

Our data are in line with those of others showing i.v. application of anti-mCD3 mAbs antibodies differentially depletes Tregs and other T cell populations [10].

### A single i.v. injection of ATG prolongs survival of heart allografts in BALB/c huCD3Ɛ mice

**Fig 5:**
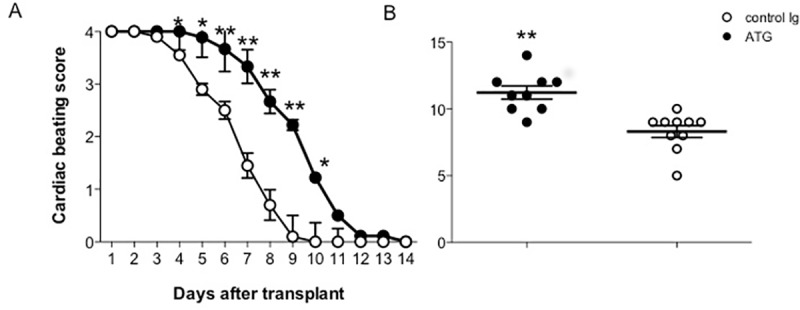
Murine cervical heart allotransplantation. BALB/c huCD3Ɛ were injected i.v with ATG or control rabbit Ig. Cervical heart transplantation was performed and graft function was evaluated by daily regular heart palpation. (A) Cardiac graft function and (B) graft survival (days). Graft function was expressed as the beating score, 0 no organ function, 1: fibrillation, only visible through magnification, 2: poor or partial organ function, 3: impairment in frequency or intensity of heart beating, 4: physiological organ function). Four to five mice per group, two independent experiments were performed. Data are shown as means ± SEM. Mann-Whitney statistical test was used, *p<0.05, ** p<0.001.

Finally, we investigated the effect of ATG on the prolongation of allotransplant survival in BALB/c huCD3Ɛ mice. We used a murine cervical heart allotransplant model [19] where C57BL/6 mice *(H2*^*b*^*)* are used as donors and BALB/c huCD3Ɛ mice *(H2*^*d*^*)* as recipients. Recipient mice were injected i.v. with 100 μg of ATG or control rabbit Ig directly before surgery. As shown in Fig 5A, in BALB/c huCD3Ɛ ATG-treated mice, heart beat scores were higher and overall, grafts were functional for a significantly longer period than in control rabbit Ig-treated mice. These data show that a single low dose of ATG was sufficient to achieve this protective effect.

## Discussion

ATG has been used in clinical settings since decades for the prevention and treatment of allotransplant rejection. It is universally accepted that *in vivo* its main effect is T cell depletion, demonstrated in peripheral blood [1,2]. Though ATG is a well-known biological compound, several points still need to be addressed with regard to its mode of action in more detail. First, no investigation of ATG effects in primary and secondary lymphoid organs has been performed in humans. Thus, to date, rabbit mATG has been used as a surrogate in several studies [13,14]. Studies in the mouse model showed that T cell depletion is induced in the spleen and the lymph nodes and that Tregs are preserved after mATG treatment [14]. The effect of ATG on secondary lymphoid organs was investigated in non-human primates [3]. This work described the depletion of T cells in spleen and lymph nodes but did not address any questions regarding a potential tolerogenic effect of ATG.

A second important aspect to be considered in this context is the polyclonal nature of ATG. T cell depletion mainly occurs through reaction with the CD3Ɛ receptor; the depleting effect might, however, be perpetuated by the multitude of antibodies with other specificities contained in ATG. In our study, we elucidated the effect involving exclusively the CD3Ɛ receptor, by using transgenic mice expressing hCD3Ɛ.

A third important aspect is the route of delivery and the dose of ATG. In clinical settings, ATG is given i.v. In the literature, the main route of delivery of mATG is intraperitoneally; moreover, the frequency of administration and applied dose seem to vary between studies [13–15,24]. All these variables may be responsible for the different outcomes in experimental settings as compared to ATG treatment in humans.

In clinical settings, various regimens of ATG administration are used [25]. Our study provides new insights for a better understanding of the mechanisms of action mediating the suppressive effects of ATG using a humanized mouse model. Moreover, it suggests that T cell depletion and Treg preservation can be achieved using a single low dose of ATG which may be desired in some clinical situations.

It has been shown that *in vitro* ATG binds to other human cell types in addition to T cells [6]. The quantitative analysis of Abs specificities contained in ATG performed by Popow at al. showed that ATG only contains 0.283 μg/mg of anti-huCD3 Abs. Accordingly, one can speculate that the total amount of these Abs contained in one ATG vial available on the market (25 mg), is approximately 7 μg [6]. Since our transgenic mice express hybrid CD3-TCR complexes (incorporating both human and mouse Ɛ chains), as expected, we detected a signal using monoclonal anti- (both anti-human and anti-mouse) CD3 mAbs in FACS staining. Moreover, we found that ATG (in doses ranging from 1 to 100 μg/ml) did not induce proliferation of splenocytes *in vitro* as compared to proliferation using anti-mouse or anti-human mAbs. These results were not surprising since the minute amounts of anti-CD3 antibodies contained in ATG preparation were not able to induce the T cell proliferation *in vitro*. On the other hand, we observed a decrease of the huCD3Ɛ signal after staining with anti-huCD3 mAbs following incubation with ATG. These data suggest that ATG binds huCD3Ɛ on T cells of humanized mice.

Notably, *in vivo*, we demonstrated that a single dose of ATG depleted T cells in the circulation as well as in spleen and lymph nodes. Since assessment of lymphocyte levels outside of the blood has apparently not yet been done in humans, our results obtained in BALB/c huCD3Ɛ mice and findings of other authors obtained in primates [3] suggest that prolonged T cell lymphopenia occurs in peripheral lymphoid organs after ATG treatment.

Moreover, it was shown that injection of anti-CD3 mAbs led to elimination mainly of DP thymocytes and CD4 single-positive T cells that display low expression of CD3 [23]. However, the modest depletion of CD3^+^ T cells that we observed in the thymus did not apply to DP thymocytes. To date, there is no evidence of T cell depletion in the thymus using anti-human or anti-mouse ATG [3,14]. Our data suggest that ATG-mediated depletion of T cells may actually occur in the thymus.

While T cell numbers recovered in the blood, a modest depletion persisted in lymph nodes and spleen 35 days after injection. Moreover, our results suggest that CD8^+^ T cells are more sensitive to ATG-mediated depletion since the CD4^+^:CD8^+^ ratio was significantly increased; these data are in agreement with experimental data obtained using mATG [14]. The reconstitution that it is observed on day 7 in the blood and in the spleen may be the result of lymphocyte replenishment from the thymus and account for the lower T cell frequencies in the latter.

We also investigated the effect of ATG-mediated depletion on other subsets of T cells and found that after 24 h, ATG preferentially depleted naïve T cells, while Teffs were less sensitive to this depletion.

Furthermore, it was reported that after treatment with anti-CD3 mAbs [10], mATG [15] or antilymphocyte serum [16] Tregs were not affected. In line with these data, we also found that after 24 h, Tregs were not depleted in any lymphoid organ checked. A very modest decrease in Treg frequencies was observed only in the lymph nodes 14 and 35 days after injection. It was reported that the Fas/FasL pathway is involved in the mATG-mediated depletion of Tregs [26]. Analysis of Helios^+^ Foxp3^+^ Tregs numbers revealed that after ATG treatment there was an increase of the Helios^+^: Helios^-^ Treg ratio. This is in line with earlier studies showing that anti-CD3 mAbs may induce Helios expression in Tregs, and that the Helios^+^ Tregs produce less IFN-γ and IL2 [27]. Interestingly, it has been shown in kidney transplant recipients receiving ATG at the time of transplantation that depletion of CD4^+^Helios^+^Foxp3^+^ T cells was less pronounced compared to CD4^+^Foxp3^-^ T cells [28]. In kidney transplant patients, Bouvy et al. found [29] an increase of the percentages of Tregs 6 months following ATG treatment in comparison with Basiliximab (anti-CD25 antibody) therapy. ATG-induced Tregs were highly proliferative and functionally suppressive. Moreover, an increased percentage of Helios-induced Tregs with a demethylated *FoxP3* gene was found in ATG treated patients.

It is not completely clear why Tregs are less susceptible to anti-CD3 T cell depletion. It was suggested that the differential impact of anti-CD3 mAbs may be due to low-level expression of CD3 in CD4^+^CD25^+^ T cells compared to CD4^+^CD25^-^ T cells [30]. Other authors reported that the TCR signalling is attenuated in Foxp3-expressing cells, possibly due to impaired TCR-mediated activation of the Akt pathway [31]. Interestingly, Tregs were also shown to be more resistant to whole body irradiation-induced cell death and that there was an increase in the proportion of Tregs in irradiated mice [32]. These observations suggest differential susceptibility of Tregs to ATG-dependent cell death.

We also observed an increase of Tregs in spleen and we cannot rule out that this phenomenon may be due to the proliferation of Tregs rather then *de novo* induction as suggested by Nishio et al. [33] demonstrating *in vivo* that expansion of Tregs does not occur through conversion of Foxp3^-^ conventional T cells into Foxp3^+^ cells, but due to proliferative expansion. Moreover, Treg expansion was shown in mice protected from experimental autoimmune encephalomyelitis after mATG treatment (24).

Finally, we investigated a possible immunosuppressive effect of ATG treatment in a murine allogeneic heterotopic heart transplantation model expressing huCD3Ɛ. Mismatched (H-2^d^ C57BL/6) heart grafts were transplanted into BALB/c huCD3Ɛ (H-2^b^) recipients directly after i.v. ATG treatment. The average survival of the heart allografts was 11 days in ATG-treated mice compared to 8 days in rabbit Ig-treated control mice (p<0.01). Thus, a single dose of ATG significantly delayed the rejection of heart allograft in ATG-treated BALB/c huCD3Ɛ mice.

In conclusion, this study shows that: (a) BALB/c huCD3Ɛ mice are a very useful model to study T cell-targeted Abs in general and ATG in particular; (b) the minute amounts of anti-CD3 antibodies contained in ATG preparation deplete T cells in the circulation and the depletion persists in lymphoid organs 35 days after ATG treatment; (c) there is a relative enrichment of Tregs as a result of lesser susceptibility of Tregs to depletion; (d) T cell depletion and an increased Tregs:non-Tregs ratio are the two mechanism that probably prolong the allograft survival.

We find it of clinical interest that within the array of broad specificities of polyclonal antibodies contained in ATG, the fraction reacting with CD3 has such an impressive immunoregulatory effect.

## Supporting information

S1 Fig Absolute cell numbers of mice injected with ATG or control rabbit Ig. BALB/c huCD3Ɛ were injected i.v with ATG or control rabbit Ig. The graphs show the absolute numbers at the given time points of (A). mCD3^+^huCD3^+^ cells in spleen (SP), lymph nodes (LN) and thymus, (B). CD4+ and CD8+, Teff and naïve T cells in the SP and LN (C). Foxp3^+^ cells in the SP and LN. Six to nine mice per group (each data point represents an individual mouse), 2–3 independent experiments were performed. Data are shown as means ± SEM. Mann-Whitney statistical test was used.
(TIFF)

S2 Fig Depletion of T cell subsets after ATG treatment, 35 days post injection. BALB/c huCD3Ɛ were injected i.v with ATG or control rabbit Ig. Cells were stained and depletion of T cells was assessed by FACS in blood, spleen (SP), lymph nodes (LN) and thymus, at the given time points. (A). Dot plots showing mCD3^+^ huCD3^+^ staining as % lymphocytes, (B) Gated on huCD3^+^, CD4^+^:CD8^+^ ratio is shown, (C) Frequencies of Foxp3^+^ Tregs shown as % lymphocytes. Four to five mice per group (each data point represents an individual mouse), 1 independent experiment was performed. *t* test or Mann-Whitney statistical test was used. Data are shown as means ± SEM.
(TIFF)
