# Supplementary figures and images for: Differential depletion of total T cells and regulatory T cells and prolonged allotransplant survival in CD3Ɛ humanized mice treated with polyclonal anti human thymocyte globulin
Source: PLoS One. 2017 Mar 3;12(3):e0173088. doi: 10.1371/journal.pone.0173088 (PMC5336254; doi:10.1371/journal.pone.0173088)

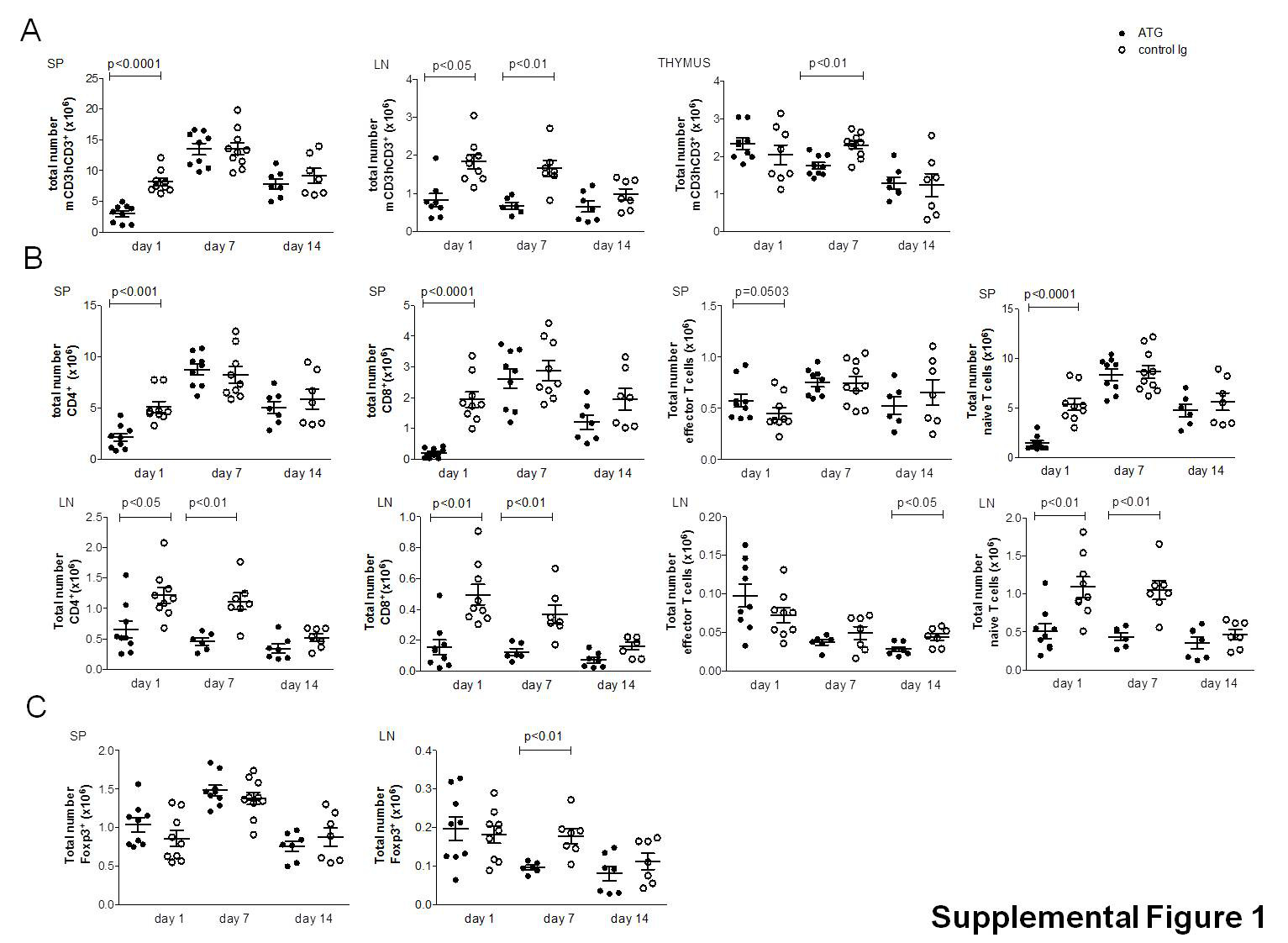

Supplement: S1 Fig — BALB/c huCD3Ɛ were injected i.v with ATG or control rabbit Ig. The graphs show the absolute numbers at the given time points of (A). mCD3+huCD3+ cells in spleen (SP), lymph nodes (LN) and thymus, (B). CD4+ and CD8+, Teff and naïve T cells in the SP and LN (C). Foxp3+ cells in the SP and LN. Six to nine mice per group (each data point represents an individual mouse), 2–3 independent experiments were performed. Data are shown as means ± SEM. Mann-Whitney statistical test was used. (TIFF) [file pone.0173088.s001.tiff]

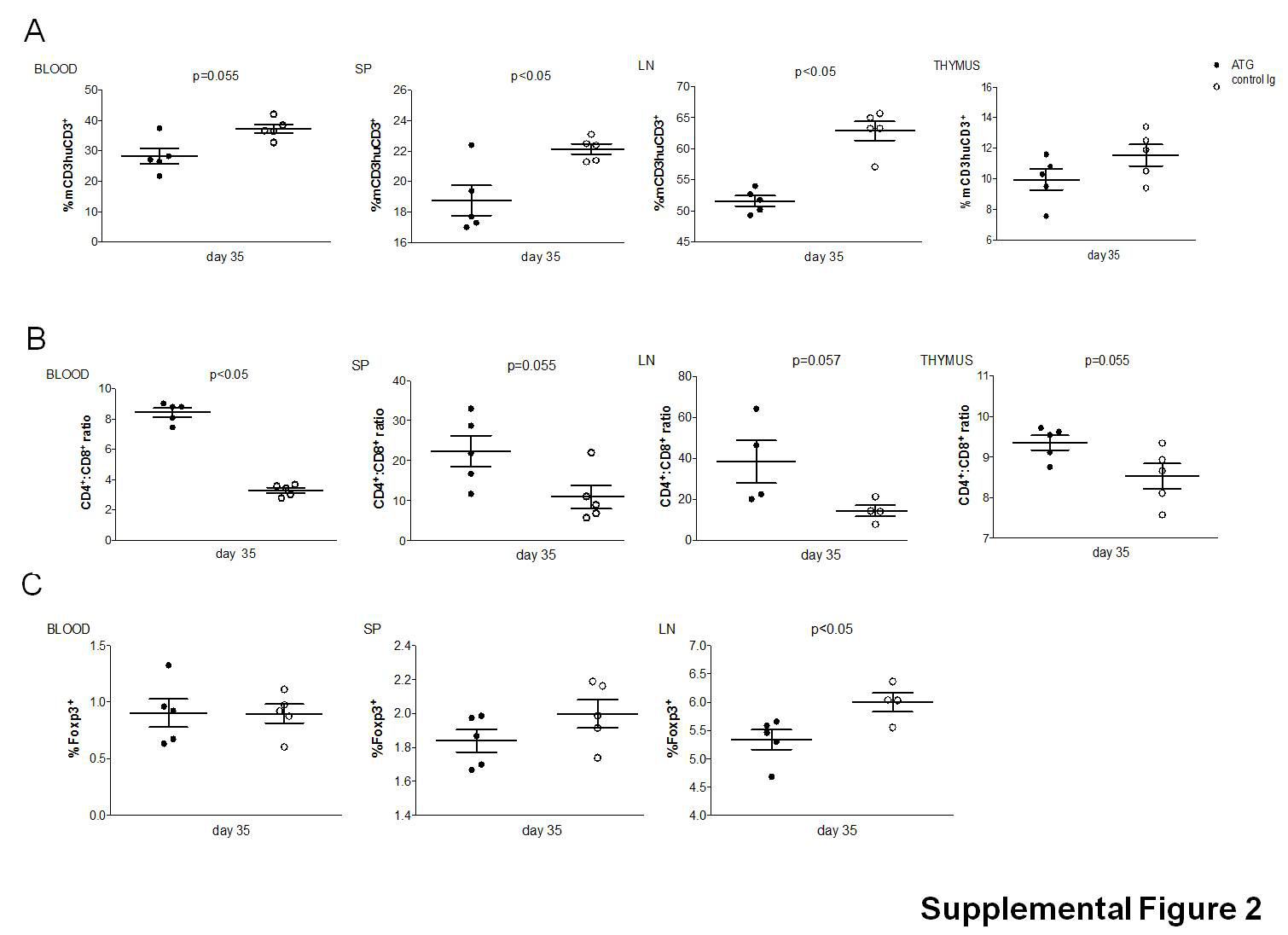

Supplement: S2 Fig — BALB/c huCD3Ɛ were injected i.v with ATG or control rabbit Ig. Cells were stained and depletion of T cells was assessed by FACS in blood, spleen (SP), lymph nodes (LN) and thymus, at the given time points. (A). Dot plots showing mCD3+ huCD3+ staining as % lymphocytes, (B) Gated on huCD3+, CD4+:CD8+ ratio is shown, (C) Frequencies of Foxp3+ Tregs shown as % lymphocytes. Four to five mice per group (each data point represents an individual mouse), 1 independent experiment was performed. t test or Mann-Whitney statistical test was used. Data are shown as means ± SEM. (TIFF) [file pone.0173088.s002.tiff]
